# Characterization of Acute Lymphoblastic Leukemia Subtypes in Moroccan Children

**DOI:** 10.1155/2009/674801

**Published:** 2009-07-19

**Authors:** Fatima Bachir, Sanae Bennani, Ali Lahjouji, Siham Cherkaoui, M'hamed Harif, Mohamed Khattab, Ilham Nassereddine, Saadia Zafad, Rajae El Aouad

**Affiliations:** ^1^Laboratoire de cytométrie en flux, Institut National d'Hygiène, Rabat, Morocco; ^2^Service d'hématologie et oncologie pédiatrique, Hôpital 20 Août, Casablanca, Morocco; ^3^Unité d'hémato-oncologie pédiatrique (UHOP), Hôpital d'enfants, Rabat, Morocco

## Abstract

We present the incidence and the immunologic characteristics of acute lymphoblastic leukemia (ALL) subsets in Moroccan children. We studied 279 unselected patients below the age of 18 years with newly diagnosed ALL. Cases were classified according to immunophenotype: 216 (77.42%) precursor B-cell phenotype (pB-cell), mature B-cell in 4 (1.43%), and T-cell in 59 (21.15%) cases. The subclassification using the CD10 antibody revealed 197 cases pB-ALL CD10+ (91.2%) and 9 cases T-ALL CD10+ (19.2%). The age distribution showed a peak in incidence between 3 and 5 years among the pB-cell ALLs subtype. There was a significantly higher frequency of males in the T-ALL subset (M/F ratio: 2.93 : 1) and more females in the T-ALL CD10+ subset when compared with the T-ALL CD10– subset. All tested pB-cell-lineage ALLs expressed CD19, CD79a, and surface CD22, terminal deoxynucleotidyl transferase (TdT) was detectable in 89.9% of cases, and cells in 74.1% of cases express CD34. All tested T-lineage ALL cells have surface CD7 and cytoplasmic CD3 (cCD3) antigens, CD5 was found in 98.2% cases, and 70.5% express TdT. CD1a, surface CD3 (sCD3), and CD4 are detected in more than 80% of cases; this frequency is higher than the 45% generally observed. Myeloid antigens occur more frequently and were expressed in 124 (57.4%) of pB-cell-ALL cases and 20 (33.9%) of T-cell ALL cases. Our results show that the distribution of ALLs in Moroccan children is similar with the general distribution pattern in developed countries except for the high frequency of T-ALL phenotype. The phenotypic profiles of our patients are close to those reported in literature for B-lineage ALLs; for the T-cell ALL subgroup, the blast cells express more CD1a, surface CD3, and CD4 while expressing less TdT. The high frequency of CD1a expression resulted in an excess of the common thymocyte subtype.

## 1. Introduction

Acute lymphoblastic leukemia (ALL) is the most common form of leukemia in children; 75% of cases occur in children under six years of age [1]. The peak in incidence is between the ages of two and five, and the vast majority of ALL cases (80%–85%) are of precursor B-lineage [1, 2]. Approximately 80% of children with B-ALL appear to be cured [1].

In Morocco, there are two dedicated pediatric oncology units, and compared to that seen in developed countries, treatment is less successful [3, 4]. The survival rate after treatment for ALL children in Morocco is less than 40%, even in specialized centers [3]. In 2003, the St. Jude International Outreach Program became a partner with the two pediatric oncology units to improve the outcome of Moroccan children with malignancies. In ALL, causes of treatment failure including abandonment of therapy, suboptimal supportive care and lack of uniform treatment guidelines [4, 5] appeared to be the most common. However, there was a possibility that the subtypes of pediatric ALL in Morocco could include an excess of high-risk leukemia subtypes. 

Since December 2003, the National Hygiene Institute laboratory has become the referral flow cytometry service for acute leukemia for the two pediatric oncology units. The main goal is to have a better survival rate by developing a specialized multidisciplinary care teams and the introduction of a uniform national treatment protocol (MARALLl 2006).

To determine the distribution of ALL subtypes in these two pediatric oncology units, we reviewed the results of cases submitted for immunophenotypic analysis.

## 2. Patients and Methods

### 2.1. Patients

From October 2003, to May 2007, 279 consecutive children (114 females, 165 males) below the age of 18 years with newly diagnosed ALL were recruited from the Onco-haematology-Pediatric service of the University Hospitals of Rabat and Casablanca. These hospitals are the main referral pediatric centers for leukemia patients in Morocco [6, 7].

The diagnosis of ALL was based on morphologic, cytochemical criteria of the French-American-British (FAB) Cooperative Working Group and flow cytometric analysis. Using these criteria, 144 cases were classified as L1, 101 cases as L2, 4 cases as L3. 30 cases were not classified as FAB subtypes.

### 2.2. Flow Cytometry

The immunophenotyping was performed at the National Hygiene Institute laboratory on bone marrow aspirate or peripheral blood samples collected in EDTA according to a two-step strategy using panels of monoclonal antibodies based on the European Group for the Immunological Characterization of Leukemia (EGIL) [8] and St. Jude Children's Research Hospital strategies [9]. Immunophenotypic characterization consisted of two consecutive steps (Table 1). According to the results obtained at the initial screening, the second level of investigation was assessed. The second panel is designed to identify stage of differentiation, prognosis features or aberrant phenotypes for monitoring minimal residual disease [10, 11].

The reactivity with fluorescent conjugated monoclonal antibodies directed against lymphoid and myeloid associated antigens was evaluated on the surface of leukemic cells. The intracytoplasmic Immunoglobin (Ig), CD3, CD79a and myeloperoxidase (MPO) antigens, as well as nuclear terminal deoxynucleotidyl transferase (TdT) staining, were evaluated by fluorescent conjugated monoclonal antibodies after fixation and permeabilization of leukemic cell. Stained cells were analyzed by flow cytometry on a three-color FACSCallibur flow cytometer (Becton Dickinson Immunocytometer Systems) which was calibrated with a set of standardized beads (Becton Dickinson Calibrate 3). Blasts initially were gated for analysis by using CD45 versus side scatter, according to the gating strategy [12].

Leukemic samples were considered positive for a particular antigen if 20% or more of leukemic cells reacted with a particular antibody.

### 2.3. Immunological Classification and Nomenclature

The cases were classified in three main ALL immunological subtypes: mature B-cell ALL, precursor B-cell ALL (pB-cell ALL), and T-cell ALL [1, 13]. A case was considered mature B-cell ALL if the cells expressed surface immunoglobulin (sIg) with Kappa or lambda light chains. Precursor B-cell ALLs include 3 immunological subtypes' early pre-B (CD19+, CD22+, CD79a and lack cytoplasmic immunoglobulin *μ* (c*μ*) and surface immunoglobulin), pre-B (CD19+, CD22+, CD79a+ exhibit cytoplasmic immunoglobulin *μ* without detectable surface immunoglobulin) and transitional pre-B (CD19+, CD22+, CD79a and express both cytoplasmic immunoglobulin *μ* and surface immunoglobulin) without Kappa or Lambda light chains [14]. T-cell ALL can also be separated into 3 maturational stages, defined as follows: proT (cyCD3+, CD7+, CD1a−, sCD3−) common thymocyte (cyCD3+, CD7+, CD1a+) and mature thymocyte (cyCD3+, CD7+, CD1a−, sCD3+).

### 2.4. Statistical Analysis

Association between the incidence of immunologic phenotype and clinical and biologic features was examined and tested by chi-square test using the Excel Stat software.

## 3. Results

The study group comprised 279 newly diagnosed and untreated acute lymphoblastic leukemia cases (<18 years). There were 114 females and 165 males with a male/female ratio of 1.45 : 1. Immunophenotypic analysis showed that the precursor B-cell phenotype was encountered in 216 (77.42%), mature B-cell in 4 (1.43%) and T-cell in 59 (21.15%) cases. The patients' characteristics are summarized in Table 2. The distribution of immunological ALL subtypes is clearly influenced by clinical features. Compared with pB-cell ALL, male sex (*P* = .008), older age (*P* = .048), higher leukocyte count (*P* < .0001) and mediastinal mass (*P* < .0001) seem to be the typical features of T-cell ALL subtype.

**Figure 1:**
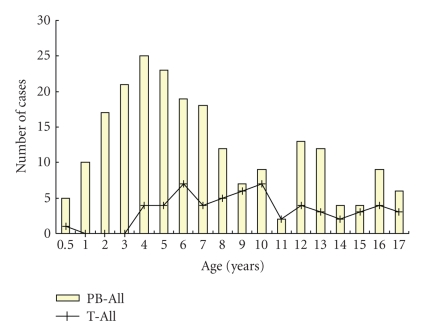
Age distribution of 275 children with acute lymphoblastic leukemia (ALL).

The age distribution of the different ALL subtypes (Figure 1) shows that pB-cell ALLs patients were younger with peak in incidence between 3 and 5 years. There was a trend for patients with T-cell ALL to have a more advanced age.

### 3.1. B-Cell-Lineage ALLs

#### 3.1.1. Precursor B-Cell

216 children with pB-cell phenotype were studied. There were 99 females and 117 males with a male/female ratio of 1.18 : 1 and median age of 6 years.

All tested pB-cell-lineage ALLs expressed CD19. Almost all cases have cytoplasmic CD79a and surface CD22, the CD20 is present in more than one-half (54.2%) of cases tested for this antigen. CD10, terminal deoxynucleotidyl transferase (TdT), HLA-DR, and CD45 were detectable in 91.2%, 89.8%, 98.1%, and 87% of cases, respectively, and cells in more than 74% of cases express CD34. In 40% of CD34 positives cases, CD45 is negative or weak. CD13 and/or CD33 occur more frequently (57.4%). CD7 is also present in few cases (3.2%).

Immunologic subclassification of 58 pB cell ALLs cases tested for cytoplasmic and surface immunoglolobin since August 2006 revealed 40 early pre-B (69%), 16 pre-B (27.6%), and 2 transitional pre-B (3.4%).

#### 3.1.2. B-Cell ALL

Four cases with L3 morphology by the FAB criteria were classified as B-cell ALL. All of them were males with ages 5, 6, 9, and 13 years. The blasts were CD19+, CD22+ in all cases and showed dimly expression of CD10. CD45 was bright, with HLA-DR positivity in all cases. The surface light chain immunoglobin expressed was Kappa in one case and lambda in 3 cases. The leukemic cells were CD34− and TdT− in all cases.

### 3.2. T-Cell-Lineage ALLs

Fifty nine children with T-cell ALL were studied. There were 44 male and 15 female (male/female ratio 2.93 : 1) with ages ranging from 0.5 to 17 years and median age of 9 years.

Based on their reactivity with various anti-T-cell monoclonal antibodies (Table 3), all tested T-cell ALL cells have surface CD7 and cytoplasmic CD3 (cCD3) antigens. CD5 was found in 98.2% cases and 70.5% express TdT. CD1a, surface CD3, and CD4 are detected in about 80% to 83% of cases. CD10 was found in 10 cases (21.3%) and absent in 37 (78.7%) of 47 T-cell ALLs cases tested for CD10. Except for 2 cases, none of the B-cell associated antigens was expressed on any T-cell ALLs cases.

Myeloid antigen expression CD13 and/or CD33 was examined, and 33.9% of cases tested positive for at least one of the two markers, more frequently CD13 than CD33, and two cases expressed the two myeloid antigens.

The immunologic subtypes, defined according to the expression of CD1a and CD3, were as follows: 3.6% pro-T, 80.4% common thymocyte, and 16.1% mature thymocyte.

### 3.3. CD10 Expression

The correlation of CD10 positivity with age, leukocyte count at presentation, gender, and FAB morphology at presentation was analyzed (Table 4). CD10 expression was found in 197 cases of pB-ALL (91.2%) and 10 cases of the T-ALL (21.3%). CD10 expression in pB-ALL was frequent in patients with age range 1–10 years and having low leukocyte count (*P*, not significant). In contrast, children with pB-ALL CD10− phenotypes were more frequently older than 10 years and have a high leukocyte count (*P*, not significant).

CD10 positivity was less common in T cell lineage and was associated with female sex and lower leukocyte count. The lack of CD10 in cases of T cell ALL was significantly (*P* = .038) associated with patients between 1 and 10 years. 

### 3.4. Lineage Heterogeneity

Lineage heterogeneity was found in both B-cell and T-cell lineage ALL with 144 (52.4%) cases having one or two myeloid associated antigens (CD13, CD33) expressed on their blasts cells. Most of them were of pB lineage (*n* = 124), T-lineage about (*n* = 20). The most common antigen expressed was the CD13 marker which was present on 100/216 (46.3%) pB-cell ALL and 13/59 (22%) T-cell ALL cases.

In nine cases (3.3%) lymphoid antigens (CD7, CD19, and CD22) were expressed. Two T-cell ALL patients' cells expressed CD19 or CD22 and cells of seven pB-cell ALL cases expressed CD7. The leukemic blast of two pB-cell ALL cases expressed also CD13 and CD33 with CD7.

## 4. Discussion and Conclusion

The present work gives the first large series of unselected acute lymphoblastic Leukemia, providing an idea about the distribution of ALL subtype in Moroccan children. Almost the majority of children with ALL in Morocco are treated at pediatric centers (Onco-haematology-Pediatric services of University Hospital of Rabat and Casablanca) [6, 7], and about 92% had a three color cytometric immunophenotyping performed at the National Hygiene Institute laboratory. 

Immunophenotyping was used in order to analyze retrospectively 279 children with acute lymphoblastic leukemia. The panel of markers proposed considers two steps; it should lead to cost saving without loss of valuable information [9]. With the first panel we were able to identify the lineage of over 97% of childhood acute leukemia. Only less than 3% were assigned to definite lineage after staining with the second panel.

The relative frequencies of immunological subtypes in this study are very similar to that reported by the developed countries of Europe and USA, except for the higher frequency of T-ALL (21.15%) [15–19]. One study carried out on 100 Moroccan paediatric patients showed a difference in the incidence of T-ALL [20]; the relative incidence of T-ALL is decreasing from 38% to 21%. The absence at that time of a system of immunophenotyping referrals and the small number of cases analyzed, could explain the difference in T-ALL incidence observed. In the present study we took advantage of the recent strategy of Acute Leukemia diagnosis in Morocco. Importantly, the frequency of T-ALL observed did not differ significantly when we include the cases diagnosed after the study period.

As it has been described in children [9, 14], the majority of cases were from pB-ALL (78.5%) with the dominance of early pre-B phenotype (69% of pB-ALL), followed by pre-B in 27.6% and Transitional pre-B in 3.4%, with typical peak between 3 and 5 year. The significantly higher incidence of CD10+ pB-ALL (197/275, 71.6%) observed was like that observed in developed centers [14, 21, 22]. In T-cell ALL subgroup, with 80.4%, there is an excess of the common thymocyte subtype displaying reactivity with CD1a as compared with the frequency reported by others [9, 14, 23, 24].

We confirmed the close association between T-cell phenotype with older age, male gender, mediastinal mass, and higher leukocyte count [1, 2, 14].

Data concerning the antigen expression for each class of ALL showed as has been reported in children in various developing and developed countries [9, 21, 24–27], every case of B cell lineage ALL expressed CD19, CD22, and CD79a. Cytoplasmic CD3, CD5, and CD7 were the most sensitive antigens for the T-cell lineage ALL. However, the results of the detailed immunologic markers analysis, underline some difference of pattern of immunologic marker expression of T-cell ALL. They show that frequencies of CD1a, surface CD3, and CD4 are higher than generally observed (less than 45%) [9], and the expression of TdT is observed in fewer cases (70.5%) than reported in previous studies, more than 90% [9, 24]. 

CD10 expression was detected in 197 cases (91.2%) of the 216 pB-ALL cases and in 10 T-ALL cases (21.3%) of the 47 cases tested for this antigen. Cases of pB-ALL having the CD10 expression had lower leukocyte count and the majority of cases (141/216) were between 1 to 10 years of age (*P* not significant). CD10+ T-ALL cases were characterized by lower leucocytes count in female sex (*P* not significant) and older age. Studies of the prognostic significance of CD10 expression in ALL have showed the CD10 expression in childhood B lineage ALL is associated with several favorable presenting features but is not an independent prognostic factor [13, 28–30]. In T cell lineage, the expression of CD10 was independently associated with favorable clinical outcome [14, 23]. However, for the larger subgroup of patients with T-lineage ALL, CD10 expression has no independent prognostic significance [30].

Aberrant expression of myeloid antigens (MyAgs) on acute lymphoblastic leukemia cells is a well-documented phenomenon and has no prognostic or therapeutic implications [15, 31–33] but can be used for monitoring MRD [32]. The myeloid-associated antigens CD13/CD33 used in the current study were the most commonly expressed in children ALL cases, and their incidence should be representative of overall MyAgs expression [15]. In the present study, MyAgs expression occurs in 52.7% of all cases (57.4% of pB cell ALL and 33.9% of T-ALL). This frequency is higher than in previous studies (between 6% and 35%) [15, 28, 31, 33]; however it is comparable to those reported by Khalidi et al. [21]. The differences observed are probably due to the criteria used to define a “percent positive” cell and the number of antigens tested [15, 21]. 

We can draw the following conclusions from our study: (1) we confirm the excellent practicability of the two immunophenotyping steps strategy for classification of ALLs, especially in countries with limited resources; (2) the distribution of ALLs in Moroccan children is similar with the general distribution pattern in developed countries, except for the high frequency of T-ALL phenotype; (3) the phenotypic profile of our patients is close to those reported in literature for B-lineage ALLs. For the T-cell ALL subgroup, the blast cells express more CD1a, surface CD3, and CD4 while expressing less TdT. The high frequency of CD1a expression resulted in an excess of the common thymocyte subtype.

## Figures and Tables

**Table 1:** Panel of Monoclonal Antibodies used in this study for immunophenotypic acute leukemia characterization.

	B-cell markers	T-cell markers	Myeloid markers	No specific markers
First step	CD79a, CD19, CD22	sCD3, cyCD3, CD7	CD13, CD33, anti-MPO	CD45
Second step	cytIg, sIg	CD1a, CD4, CD5, CD8	CD117, CD41, CD61, GlycoA	CD34, DR, TdT, CD10

sIg: surface immunoglobulin, cyIg: cytoplasmic immunoglobulin, sCD3: surface CD3, cyCD3: cytoplasmic CD3, MPO: myeloperoxidase, Glyco A: Glycophorin A, DR: HLA-DR: antigen, TdT: terminal deoxynucleotidyl transferase.

**Table 2:** Patients' characteristics.

Features	All patients (*n* = 275)	T-cell ALL (*n* = 59)	pB-cell ALL (*n* = 216)	*P*-value
Age at diagnosis (y)				
Median	7	9	6	
Range	0.5–17	0.5–17	0.5–17	
				.048^‡^
<1	6 (2.18%)	1 (1.69%)	5 (2.31%)	
1–10	184 (66.91%)	32 (54.24%)	152 (70.37%)	
>10	85 (39.91%)	26 (44.07%)	59 (27.31%)	
Gender				.008^‡^
M	161 (58.55%)	44 (57.63%)	117 (54.17%)	
F	114 (41.45%)	15 (25.42%)	99 (45.83%)	
Sex ratio (M/F)	1.41 : 1	2.93 : 1	1.18 : 1	
FAB subtype n (%)				.85
L1	144 (52.36%)	34 (57.63%)	110 (50.93%)	
L2	101 (36.73%)	22 (37.23%)	79 (36.57%)	
NS	30 (10.91%)	3 (5.08%)	27 (12.50%)	

Leukocyte count (×10^9^/L)				
Median	20.14	116	11.38	
Range	1–830	1.5–718	1–830	
				<.0001^‡^
<50	178 (94.96%)	16 (27.12%)	162 (75.35%)	
>50	96 (35.05%)	43 (72.88%)	53 (24.65%)	
CNS disease**	4/218 (1.83%)	2/45 (4.44%)	2/173 (1.16%)	.42
Mediastinum**	35/218 (16.05%)	28/45 (62.22%)	7/173 (4.05%)	<.0001^‡^

ALL: acute lymphoblastique leukaemia, F: female, M: male, Y: year, CNS: central nervous system, NS: not specified.

*Mature B-cell ALL excluded.

**Cases with completed data.

^‡^ *P* ≤ .05 (statistically significant).

**Table 3:** Immunophenotypic profiles of 275 de novo acute lymphoblastic leukaemia.*

Markers	T-cell ALL (*n* = 59)	pB-cell ALL (*n* = 216)**
CD1a^+^	45/56 (80.4)	—
sCD3^+^	49/59 (83.1)	0/216 (0.0)
cCD3^+^	57/57 (100)	0/216 (0.0)
CD4^+^	42/51 (82.4)	—
CD5^+^	54/55 (98.2)	—
CD7^+^	59/59 (100)	7/216 (3.2)
CD8^+^	32/52 (61.5)	—
CD10^+^	10/47 (21.3)	197/216 (91.2)
cyCD79a^+^	0/48 (0.0)	162/162 (100)
CD19^+^	1/59 (1.7)	216/216 (100)
CD20^+^	0/8 (0.0)	26/48 (54.2)
CD22^+^	1/47 (2.1)	216/216 (100)
CyIg^+^	—	18/58 (31.0)
SIg^+^	—	2/58 (3.5)
TdT^+^	31/44 (70.5)	142/158 (89.8)
CD34^+^	25/59 (42.4)	160/216 (74.1)
HLA-DR^+^	4/39 (10.3)	152/155 (98.1)
CD13^+^	13/59 (22)	100/216 (46.3)
CD33^+^	9/49 (15.3)	75/216 (34.7)
CD13^+^, CD33^+^	2/59 (3.4)	51/216 (23.6)
CD13^−^, CD33^+^	7/59 (11.9)	24/216 (11.1)
CD13^+^, CD33^−^	11/59 (18.6)	49/216 (22.7)
CD13^+^ and/or CD33^+^	20/59 (33.9)	124/216 (57.4)
CD13^−^, CD33^−^	39/59 (66.1)	92/216 (42.6)
CD45^+^	59/59 (100)	187/216 (87)

ALL: acute lymphoblastique leukaemia, −: not applicable, MPO: myeloperoxidase, TdT: terminal deoxynucleotidyl transferase, cy: cytoplasmique, s: surface, +: positive, −: negative, Ig: immunoglobulin.

*Result are given as number positive/number tested (% positive).

**Mature B-cell ALL excluded.

**Table 4:** Clinical and biological features according to CD10 expression in children with ALL.

		No. of patients (%)					
		pB-cell ALL (*n* = 216)			T-cell ALL (*n* = 47)		
Features	Total of patients	CD10−	CD10+	*P* value	CD10−	CD10+	*P* value

Gender				.92			.24
F	113	9 (9.09)	90 (90.91)		9 (64.29)	5 (35.71)	
M	150	10 (8.55)	107 (91.45)		28 (84.85)	5 (15.15)	
Leukocyte count (×10^9^/L)				.31			.81
<50	177	12 (4.71)	150 (92.59)		11 (73.33)	4 (26.67)	
>50	85	7 (13.21)	46 (86.79)		26 (81.25)	6 (18.75)	
FAB subtype				.67			.89
L1	135	10 (9.09)	100 (90.91)		19 (76.00)	6 (24.00)	
L2	98	5 (6.33)	74 (93.67)		15 (78.95)	4 (21.05)	
Age (Y)				.27			.038^‡^
<1	6	0 (0.00)	5 (100)		0 (0.00)	1 (100)	
1–10	147	11 (7.24)	141 (92.76)		20 (90.91)	2 (9.09)	
>10	83	8 (13.56)	51 (86.44)		17 (70.83)	7 (29.17)	

ALL: acute lymphoblastic leukaemia, F: female, M: male, Y: year.

^‡^ *P* ≤ .05 (statistically significant).
